# Silk-Based Materials for Hard Tissue Engineering

**DOI:** 10.3390/ma14030674

**Published:** 2021-02-01

**Authors:** Vanessa J. Neubauer, Annika Döbl, Thomas Scheibel

**Affiliations:** 1 Lehrstuhl Biomaterialien, Fakultät für Ingenieurwissenschaften, Universität Bayreuth, Prof.-Rüdiger-Bormann-Straße 1, 95447 Bayreuth, Germany; Vanessa.Neubauer@bm.uni-bayreuth.de (V.J.N.); Annika.Doebl@bm.uni-bayreuth.de (A.D.); 2 Bayreuther Zentrum für Kolloide und Grenzflächen (BZKG), Universität Bayreuth, Universitätsstraße 30, 95440 Bayreuth, Germany; 3 Bayerisches Polymerinstitut (BPI), Universitätsstraße 30, 95440 Bayreuth, Germany; 4 Bayreuther Zentrum für Molekulare Biowissenschaften (BZMB), Universität Bayreuth, Universitätsstraße 30, 95440 Bayreuth, Germany; 5 Bayreuther Materialzentrum (BayMAT), Universität Bayreuth, Universitätsstraße 30, 95440 Bayreuth, Germany

**Keywords:** silk fibroin, silk spidroin, biomineralization, composite materials, bone, teeth, cartilage, tendon

## Abstract

Hard tissues, e.g., bone, are mechanically stiff and, most typically, mineralized. To design scaffolds for hard tissue regeneration, mechanical, physico-chemical and biological cues must align with those found in the natural tissue. Combining these aspects poses challenges for material and construct design. Silk-based materials are promising for bone tissue regeneration as they fulfill several of such necessary requirements, and they are non-toxic and biodegradable. They can be processed into a variety of morphologies such as hydrogels, particles and fibers and can be mineralized. Therefore, silk-based materials are versatile candidates for biomedical applications in the field of hard tissue engineering. This review summarizes silk-based approaches for mineralized tissue replacements, and how to find the balance between sufficient material stiffness upon mineralization and cell survival upon attachment as well as nutrient supply.

## 1. Introduction

The development of hard tissue in the human body is a process of mineral formation by cellular metabolism, named biomineralization, yielding support structures of the skeleton and neighboring tissues such as tendon and cartilage or functional tissues such as teeth [1]. There are several different mineralization pathways, but they are not yet fully explored [2]. Generally, mineral formation in tissues needs to be highly controlled to prevent local over-mineralization, which could be pathogenic [2]. The high process control of biomineralization is provided by tissue-specific cells and biopolymers such as proteins, which are templating and nucleating mineral formation [3]. Therefore, biogenic crystals often exhibit a different morphology than their geogenic counterpart [4].

Tissue-specific cells are taking a crucial role in biomineralization as they trigger mineral nucleation and growth upon secretion of so-called non-collagenous proteins [2,5]. The main proteinous material (90 wt.%) of hard tissues is collagen type I as flexible filler in this composite material, while the non-collagenous proteins cover the remaining 10 wt.% [2]. Collagen is not mineralized on its own, but collagen fibrils can interact with non-collagenous proteins, which induce mineralization from saturated media at the gap regions of the stacked triple-helical collagen fibrils [2,5]. The phosphorylated, non-collagenous proteins of the so-called SIBLING family (Small Integrin-Binding Ligand, *N*-Linked Glycoprotein) include bone sialoprotein and osteopontin in bone-related tissues, whereas in teeth dentin and cementum, dentin matrix protein 1 and dentin phosphoryn are present. These proteins provide two functions, as on the one hand, they can bind at specific locations to the structural collagen scaffold and on the other hand, they can bind ions due to their, in most cases, highly charged nature with repetitive motifs of glutamic or aspartic acid residues [5]. This local charge density allows to accumulate mineral ions and, thereby, to initiate crystal nucleation, when the ion density reaches a critical concentration, which then triggers the further mineralization processes in mineralized tissues, such as bone, teeth, cartilage and tendon [5].

Further, mineralization is driven by tissue-related osteoblasts (in bone and tendon), odontoblasts (in teeth) and chondrocytes (in cartilage) upon the accumulation of ions from the surrounding environment in mostly separated membrane vesicles [6]. With ongoing mineralization, the extracellular matrix around these cells densifies, and nutrients and oxygen are increasingly provided only passively by diffusion until easy nutrient supply is finally prevented. In the case of bone and neighboring tissue, osteoblasts differentiate into osteocytes [1]. Osteoclasts, on the other hand, are constantly remodeling fully mineralized tissue to guarantee healthy and reconstructed bone [7].

For traditional and engineering approaches to reconstruct hard tissues, natural processes have to be understood. Further, as bone represents the most abundant fully mineralized tissue, a majority of tissue engineering approaches focus on respective reconstructive solutions. Bone defects such as fractures easily occur, for example due to critical non-physiologically high loads. Shortly after fracture, inflammatory responses are initiated at the defect site, followed by a cell-induced regeneration cascade for initial callus formation, which is then remodeled to form new bone. With progressing age, bones become increasingly brittle due to changes in the cellular metabolism of osteoblast cells, which is indicated by 10–40 times lower strain rates until breakage. One possible reason might be remodeling cycles, which affect the mineral phase and allow more micro-cracking, finally leading to bone failure [8]. Once fractured, bone defects can be detected by X-raying of the defect site. New techniques such as ultrasonography for detecting bone fractures are more sensitive than classical radiographs, which are typically used to trace fractures of long bones. Sonographic methods provide the advantage of no radiation exposure, lower cost and wider availability in non-hospitals. A study among German general practitioners showed that most articulated sono-methods are inferior to classical X-ray [9]. In clinical procedures, the defect site is often bridged and stabilized with bone platelets or screws to stabilize material in place during the regeneration process [10]. In order to further support bone healing or large defects with bone loss, hard tissue engineering methods are increasingly used. In contrast to the self-regenerating ability of bone tissue, other mineralized tissues rely more on artificial replacement than supportive healing.

In the following, approaches for hard tissue engineering, with the focus on bone, and some examples including teeth, cartilage and tendon, based on silk scaffolds are discussed in detail.

## 2. Tissue Engineering Approaches

**Figure 1:**
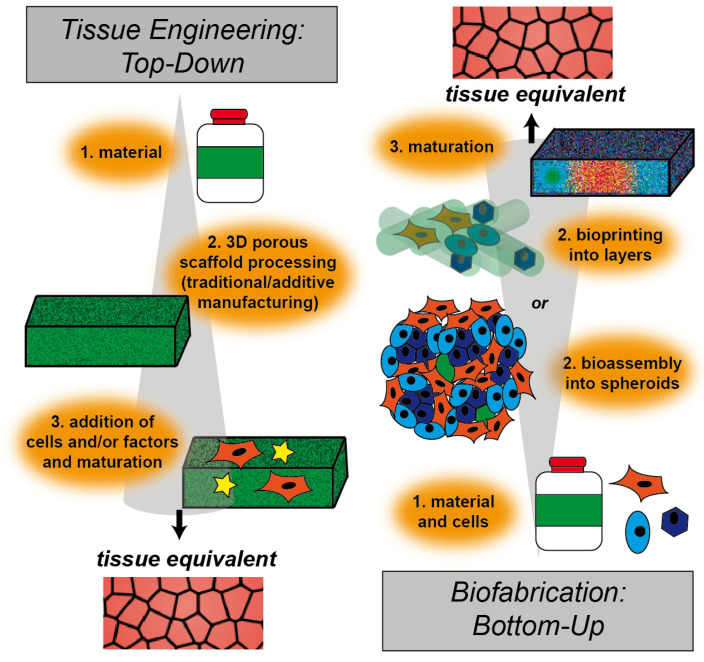
Schematic illustration of engineering approaches to fabricate tissue. In top-down strategies, a scaffold is produced, followed by cell seeding and/or addition of factors for cellular stimulation. This technique is called tissue engineering. Bottom-up approaches use cells and raw materials simultaneously to build larger constructs, which are then maturated. This technique is called biofabrication.

Defined in 1993, tissue engineering is the combination of principles of engineering and life sciences with the goal of developing biological substitutes that are able to restore, maintain or improve biological tissue function or a whole organ [11,12]. While one part of this interdisciplinary field deals with the generation of 3D models for the development of therapies, its main potential lies in regenerative medicine. With the goal of replacing tissues and organs damaged by trauma, disease or age, the ever-extending field of TERM (tissue engineering and regenerative medicine) includes basic and advanced cell and stem cell biology, scaffold material research and various fabrication and processing techniques [13,14]. The classical TERM approach to overcome drawbacks of autografts or allografts consists of scaffold-based top-down strategies (Figure 1). Such engineered tissues are typically created by manufacturing biodegradable polymeric scaffolds on which cells are seeded. During cultivation, and in some cases stimulated by perfusion, growth factors or mechanical cues, cells dynamically remodel and replace the scaffold through degradation and new extracellular matrix (ECM) deposition [15]. Traditionally, three-dimensional scaffolds are manufactured by employing techniques such as freeze-drying, leaching of particles or salt, chemical or gas foaming and thermally induced phase separation [16,17,18,19]. While these processes allow partial control over the scaffold properties, there are severe restrictions to generate precise micro-architectures, including pore size, geometry and connectivity.

With the rise of additive manufacturing, more techniques are available to create scaffolds for tissue engineering, overcoming previous restrictions. With an emphasis on the regeneration of bone tissue, four major layer-by-layer methods have been reviewed recently by Madrid et al. [20]. A variety of natural and synthetic polymers, as well as ceramics and bioceramics and even metals can be processed using stereolithography (SLA), selective laser sintering (SLS), fused deposition modeling (FDM) and three-dimensional printing (3DP). These techniques generally allow for more accurate scaffolds with better resolution. The specific processes, including laser and heat treatment, nevertheless tremendously restrict the choice of material [20]. Since additive manufacturing is based on computer-aided-design, structures that are more sophisticated can be created, including patient-specific scaffolds with the help of computer tomography. While these top-down approaches allow for good reproduction of the macroscopic structures of desired tissues and organs, the complexity, micro-arrangement and heterogeneity of natural tissues, including different cell types and materials, is far beyond what is found in such artificial acellular scaffolds [15,21].

To overcome this limitation, a multitude of bottom-up approaches has been developed in recent years. In contrast to traditional top-down approaches, where first the scaffold is produced, followed by seeding with cells, in bottom-up approaches, cells are used from the very beginning in combination with materials to build up tissue constructs step-by-step (i.e., bottom-up) (Figure 1). Biocompatible materials in various morphologies, like particles, one-dimensional fibers, two-dimensional films and three-dimensional hydrogels, have been used, alongside cells, as building blocks to generate assemblies at the nano- or micro-scale. Further self- or directed-assembly leads to engineered macroscopic three-dimensional tissue constructs. A comprehensive review examining these advanced bottom-up approaches has recently been published by Gaspar et al. [21,22].

Benefits of these strategies include the involvement of cells in the development of the tissue right from the beginning, as well as the possibility to generate constructs made from different types of assemblies, leading to various cell types and materials organized hierarchically within the resulting construct. Different assemblies can be divided into two main categories, mostly scaffold-free cell-rich and cell-biomaterial assemblies. Adhesive surfaces, possibly in combination with functionalized and/or non-adhesive surfaces, are used to generate monolayer cell sheets by cultivation and subsequent detachment of the grown layer. Stacking, rolling and folding of these monolayer sheets is the basis to create combined three-dimensional assemblies, including multicellular and pre-vascularized constructs [23,24]. By using cellular spheroids, often made of mesenchymal stem cells, as scaffold-free building blocks, processes like cell-cell and cell-ECM interactions, differentiation and fusion are recapitulated [25,26]. In addition, genetic or chemical engineering of the cell surface allows control over cellular behavior and assembly into higher-order structures [27,28]. Inclusion of biological materials is a crucial part of bottom-up tissue engineering strategies, such as the addition of biocompatible layers within cellular sheets, functionalized with nucleic acids, viruses, enzymes and structural proteins, as well as peptides and polymers. To increase structural assembly within cellular spheroids or hydrogels, fibers and particles can be incorporated. These materials can add structural support and guidance, promote and/or control the assembly of building blocks and stimulate cellular behavior in general [21,29,30]. For example, a silk fibroin derived hydrogel was used as a scaffold for articular cartilage tissue engineering, and integrated poly(lactid-co-glycolid) nanoparticles were used to simultaneously deliver two growth factors, resulting in beneficial effects on proliferation and differentiation of dental pulp stem cells [31]. On the way to tissue or organ replacement, such multicellular and multimaterial assemblies are used to generate vascularized multicomponent constructs or spatially organized multiblock hydrogels [21]. 

In the context of advanced bottom-up tissue engineering approaches, a new field called biofabrication has been reviewed recently by Groll et al. [32]. Biofabrication mainly, but not solely, uses additive manufacturing techniques to process bottom-up building blocks into hierarchically structured cell-biomaterial constructs. Biofabrication describes the automated generation of biologically functional constructs through bioprinting, meaning the direct spatial arrangement of cells, materials and/or factors, and through the automated assembly of cell-containing building blocks, so-called bioassembly. In both cases, in vitro maturation and or fusion of the products is a crucial step before obtaining a tissue equivalent for implantation or pharmaceutical screening [32]. Relevant technologies within biofabrication have been recently reviewed by Moroni et al. [33]. With the possibility of simultaneous deposition of cells and material in an additive manufacturing process, bioplotting, ink-jet bioprinting and valve-jet bioprinting are major biofabrication tools for bottom-up tissue engineering and regenerative medicine. Formulations of materials, cells and biological molecules, so-called bioinks, are processed using these technologies. Bioplotting, also called robotic dispensing or extrusion bioprinting, dispenses continuous filaments of hydrogel materials or bioinks through a nozzle (piston-, screw-, or pneumatic-driven). Droplets are ejected over a nozzle head, controlled either by piezo- and thermal-actuators (ink-jet) or by solenoid micro-valves (valve-jet) [32,33].

All approaches, whether they include manufacturing a scaffold followed by cell-seeding or bioprinting/bioassembly, have strict requirements on the used material. Physical and mechanical properties need to be suitable for processing using the respective technology on the one hand and ensure cellular survival and proliferation on the other. The material also plays an important role in guiding specific cellular development and maturation, for example, by surface functionalization, the inclusion of biological molecules or the tuning of degradation behavior. With the goal of implantation of constructs, biocompatibility, meaning the performance of intended purpose without evoking an immune response, is absolutely required and can be enhanced e.g., upon introduction of nanoparticles [34,35,36]. Due to their inherent biological and chemical similarities to native tissue, natural polymers, natural polymer-based composites and bioceramics are of great interest for tissue engineering applications. Due to the high load-bearing requirement, hard tissue engineering approaches so far mainly focus on top-down strategies using porous scaffolds for cell seeding [37,38].

## 3. Hard Tissue Engineering

### 3.1. State of the Art

After diagnosis of a bone defect, the respective site is commonly deprived from extensive movement as both bone sides need to reconnect during regeneration in a correct manner, otherwise malfunction might be the result of improper healing. The origin of the cells, which are taking part in bone repair, were found to influence the healing progress. The cells present in bone encompass, for example, stem cells during bone healing or endothelial cells building vasculature, but also pre-osteoblasts, which differentiate into osteocytes during bone formation and maturation as described above. Osteoclasts are undertaking the function of degradation, which is a continuously ongoing process to maintain healthy bone and allow for expansion of the skeleton during the development of children [39]. When artificially delivered into bone defects, neural crest-derived frontal bone and mesoderm-derived parietal bone cells from newborn rats were found to exhibit both similar bone regeneration ability, although the mesodermal cells showed a potentially higher bone regeneration efficiency in vitro [40]. MC3T3 E1 pre-osteoblast cells were posed in hydroxyapatite microcracks similar to bone fractures and found to underlie initial apoptosis at a region of 200 nm around the cracks [41]. Besides fixation, flexoelectricity, meaning the ability to generate electricity under pressure, was found crucial for bone healing [41]. Exposed to strain such as physical activity during bone healing, bone regeneration was increased, and so rehabilitation measures actively contributed to tissue regeneration [42]. With near-infrared fluorescent probes [43], bone repair could be imaged concerningin vitro differentiation of human mesenchymal stem cells into osteoblasts. A cyclic peptide coupled with a fluorophore was used to bind to α5β1 integrin as an osteoblast-specific marker. A second probe was coupled with the drug pamidronate to a fluorescent gold nanocluster, where the drug bound specifically to hydroxyapatite and allowed for monitoring osteogenesis [43].

**Figure 2:**
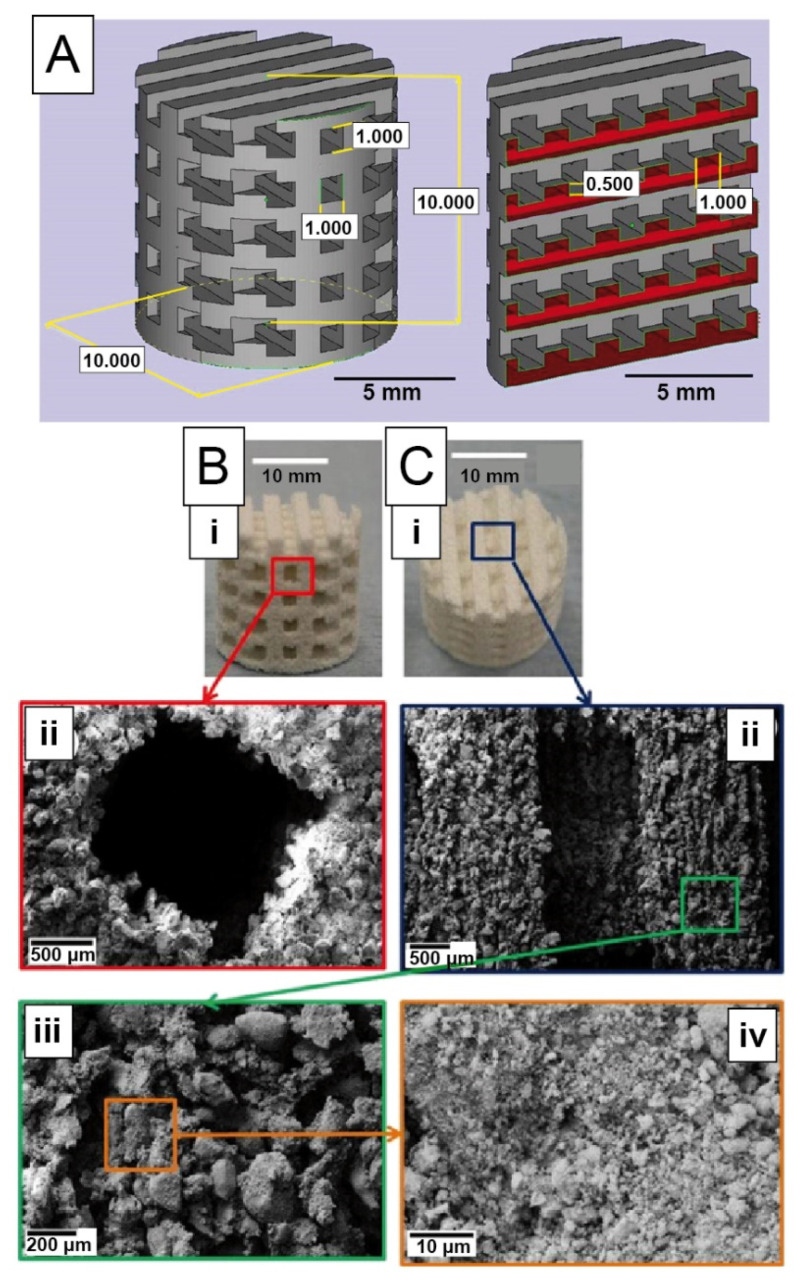
Scaffolds containing 50 wt.% hydroxyapatite nanoparticles suspended in poly(vinyl alcohol) as matrix material. (**A**) CAD design of a layered scaffold showing porous structures in the cylinder. Extrusion printed scaffolds after drying and thermal curing in side-view (**B**(**i**)) with higher magnification of a channel pore (**B**(**ii**)), and in top view (**C**(**i**)) with higher magnification of a channel (**C**(**ii**)), showing individual hydroxyapatite particle agglomerates (**C**(**iii**)**,C**(**iv**)). Reprinted and adopted with permission from ref. [50]. Copyright 2015 Elsevier.

Loss of bone material due to cancer or other pathogenic relations such as osteoporosis is often not recovered spontaneously and needs tissue replacement. Autologous bone grafts are still considered as the gold standard transplant due to facilitated integration at the defect site. As concerns about donor availability, healing and disease transmission arise, artificial bone substitutes become increasingly attractive to overcome these obstacles [39]. Therefore, titanium implants are state of the art as they are biologically inert materials, which offer high load transmission. Unfortunately, these foreign body materials are rarely fully integrated into the surrounding tissue and might become loose; therefore, surgical rearrangement might be necessary. One major reason for this issue is a bacterial infection, especially concerning dental implants with extensive biofilm formation [34]. To improve integration, for example titanium alloy (Ti6Al4V) implants with TiO_2_ nanotubes were coated with silk fibroin, which was found to enhance osteoconductive and osteogenic properties in case of bone implant performance [44]. Bone, cell and implant interaction was found to be enhanced for MG63 bone cells and human mesenchymal stem cells, which is beneficial for implant applicability. Biomimetic minerals for hard tissue engineering, which enhance osseointegration, can rely on biosimilars such as calcium sulfate or phosphate ceramics as synthetic and hydroxyapatite as a naturally occurring form of bone mineral [39]. Building scaffolds out of these materials can be realized upon melting and fusing individual ceramic particles using laser sintering at temperatures above 1000 °C [45,46,47]. Utilizing this rapid prototyping technique, also polymeric carrier materials can be fused at lower temperatures (about 70–200 °C) whilst molding and binding ceramic particles into bionanocomposites and simultaneously removing the binder [48,49]. With such polymeric binders, 3D extrusion and additional sintering of the composite materials is possible, yielding solely the remaining solid ceramic structures (Figure 2) [50,51].

Further, injectable calcium phosphate cements including ceramics and a curing agent were invented by Brown and Cho in the 1980ies to fill dental cavities in the first place [52]. As state of the art, synthetic polymeric materials are widely used as matrix materials in hard tissue engineering, however, they often cannot complement features of biomaterials such as non-toxic degradation products and bioactive surfaces for cell adhesion [53].

### 3.2. Design Criteria and Challenges

**Figure 3:**
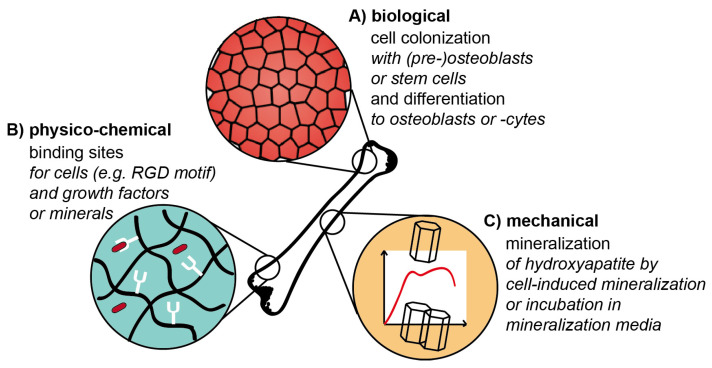
Illustration of biological, mechanical and physico-chemical factors of scaffold materials relevant for hard tissue engineering. (**A**) Media, growth factors, etc. are needed for tissue-specific cell colonization and differentiation on artificial scaffolds. (**B**) Materials/scaffolds have to provide binding sites for cells, factors and minerals. (**C**) Biomineralization is necessary to gain composite materials with adopted mechanical properties (such as stiffness, etc.).

It is important that various design criteria and factors have to be taken into account in tissue engineering approaches to fulfill the requirements of a successful tissue engineering construct (Figure 3). In the case of hard tissues, besides biological and physico-chemical cues, also the appropriate mechanics play an important role. In the following, these aspects are discussed in more detail and illuminated why they can be challenging during scaffold preparation.

Concerning the mechanical design, it has to be taken into account that mature bone has compressive strengths in the order of up to 20 GPa [54], whereas they are far lower for immature bone, as the mineralization process is still ongoing [55,56]. However, not only high strength but also flexibility must be provided. Therefore, mostly brittle materials are not suitable for bone regeneration applications, as the risk of failure is high [8]. It can be challenging to combine high load-bearing materials with high flexibility, but these mechanical requirements can be fulfilled in biomaterial matrices applying reinforcing filler materials such as ceramic particles into composite materials. To gain homogenous mineralization, it is important that filler and matrix material interact well with each other to avoid phase separation, which is an additional criterion. Practical hints can be found when taking a closer look at the natural blueprint: Bone is a composite material [57] with collagen fibrils (20–30 wt.%) and ceramic particles made of hydroxyapatite (60–70 wt.%) [2]. Besides composite materials, biomineralization of protein precursor materials can be triggered in vitro upon immersion in mineralization agents forming calcium phosphate species. These can for example be single aqueous salt solutions, which are subsequently applied to the materials [58,59,60]. More complex mineralization is provided by Simulated Body Fluid, a model solution at pH 7.4, which was designed to simulate mineralization processes found during bone formation. Its ion composition and concentration are proximately close to human blood plasma [61].

Tailoring mechanical properties upon controlled mineralization is highly interconnected with the scaffold’s biological function and vice versa. During mineralization of tissue, cells play an important role as they secrete non-collagenous proteins with highly located charge [2]. Especially SIBLING proteins [5] are to be mentioned among others, as they coordinate nucleation, growth and inhibition phase during mineral formation as they accumulate ions from the surrounding intestinal fluids [62]. Further, hydroxyapatite precursor phases can be accumulated in cell membrane-bound vesicles and released at mineralization sites [6]. As a result, tissue-specific cell colonization is an additional design cue to mimic natural tissue in engineered constructs. Its respective challenge is posed not only by cell adhesion to the surface or in the construct but also to trigger osteoblast lineage in osteoblast precursor cells or stem cells. Biomineralization and osteogenic differentiation were found to be highly dependent on matrix stiffness [63]. 2D surfaces of different controllable substrate stiffness showed the best results for medium stiffness (50–100 kPa), as mineralization was completed after three weeks. Osteoblast differentiation was directly related to the formed mineral layer and only indirectly regulated by matrix stiffness [63]. The release of ions from the material, which is sensed by cells, can also lead to differentiation responses. One example for such materials is 45S5 Bioglass embedded in silk fibroin/gelatine scaffolds [64]. The bioglass composition comprises SiO_2_, CaO, Na_2_O and P_2_O_5_, and the ion release profile triggers osteogenic cell differentiation [65]. For this functionalization, it is important to control the osmotic balance of the media for cell survival.

Moreover, not only mechanical but also physico-chemical properties of the scaffold can lead to desired cell differentiation. The design of such cues can be related to binding sites for cells, growth factors or minerals. Besides cell-specific binding motifs [66], the integrin binding peptide motif arginyl glycyl aspartic acid (RGD) [67] is universally applied. The incorporation of this motif can be a challenge when it is not intrinsically provided by the biomaterial. This can be solved upon genetic engineering of proteins used in the material or chemical coupling of the motif to the material [68]. Related to the natural tissue, growth factors such as the most important one, the Transforming Growth Factor beta (TGF-beta), as well as bone morphogenetic proteins are agreed to have a beneficial impact on the success of hard tissue engineering scaffolds [69]. The factors can be delivered via the construct and trigger stem cells towards osteo-differentiation [70]. As a challenge, their concentration must be maintained [69] at levels confirmed to be active (nM) during cell cultivation by specific binding, otherwise, scaffolds become depleted fast by diffusion. Binding sites for ions were discussed above to be provided by non-collagenous proteins with located charges. Mimics of these proteins can be designed and incorporated into the scaffold. However, the preparation of hybrid proteins from silk and non-collagenous proteins can be challenging to gain functional mineral binding sites [71,72].

Taking all these complex requirements into regard, scaffolds must comply not only with mechanical but also physico-chemical and biological demands to build a successful hard tissue engineering construct. One crucial role plays the material choice. The named requirements can be met, for example, with synthetic or natural materials [14]. However, synthetic materials pose the risk of toxic degradation products during tissue regeneration, and their biocompatibility is limited [73]. Naturally derived materials avoid these obstacles. Further, they naturally can provide biological and/or mineral binding sites. However, disease transmission from donor animal sources and material heterogeneity must be avoided. Collagen and gelatin are common materials due to their native occurrence in bone and the presence of biomineralization nucleation sites. Moreover, bone takes a long time to develop, and the collagen will often degrade before it can be remodeled. Among artificial natural biomaterials, silk appears to be an attractive material, as it provides non-toxicity and biodegradability. Further, silk proteins can be produced biotechnologically, modified and processed into a variety of morphologies [73]. The upcoming sections will shed light on how silk materials can be used, for example, as matrix materials for bone tissue engineering.

## 4. Silk

### 4.1. Naturally Derived Silk

Silks are a class of protein fibers spun by arthropods such as fleas, mites, spiders and silkworms, amongst others. They are based on fibrous proteins containing highly repetitive amino acid sequences stored in the animal as liquid and transformed into fibers once shear stress is applied during spinning [74].

Fibers produced by the silkworm *Bombyx mori* consist of two silk fibroins (SF) and glue-like (non-silk) proteins named sericins. The fibroin heavy chain consists of a highly repetitive (12 times) glycine and alanine-rich region and two hydrophilic N- and C-terminal domains. The fibroin light chain is an arginine- and lysine-rich non-repetitive protein [75]. Upon secretion, both fibroins and a third small glycoprotein, p25, assemble into twin filaments that represent the inner part of the core-shell structure typical for *B. mori* silk. Sericins coat and stick these fibroin filaments together. The structure is completed by an additional coating with various proteins for the protection of the cocoon [76].

The most commonly studied spider silk is dragline silk made of proteins secreted from the major ampullate gland, and it consists of multiple proteins, called spidroins. The overall layout and amino acid composition of these major ampullate spidroins (MaSps) are similar to the architecture of the fibroin heavy chain. The primary structure (i.e., amino acid composition) of the core domains, however, is quite different. One spidroin filament is coated with a thin shell containing other silk proteins, lipids and glycoproteins constituting a core-shell-structure [77]. While most silk fibers have a high toughness compared to man-made fibers, spider silk outperforms the others concerning its mechanical properties [75].

Natural spider silk fibers, mostly from female adult *Nephila* spiders, have been used as suture threads or processed into scaffolds for neuron guidance, skin repair and bladder reconstruction [78]. While most spiders exhibit cannibalistic behavior, silkworms can easily be farmed to harvest their silk in large quantities [79]. Consequently, silk from the domesticated silkworm *B. mori* has been extensively characterized and is the main silk material used in biomedical applications, for example, as sutures and in tissue engineering and regenerative medicine approaches [38]. To extract fibroin from the harvested cocoons, a thermochemical treatment is applied, called *degumming*. This step is particularly important since it also removes the sericin component from the fibroin fibers, which has been shown to be problematic by causing immune reactions [80].

### 4.2. Bioengineered Silk

Advanced tissue engineering approaches cannot only take the physical properties of the fabricated scaffold into account. Apart from biocompatibility, the degradation rate of specific scaffolds is highly important. In the best case, the degradation should be identical to the rate at which new tissue is formed by the cells. When working with *B. mori* silk fibroin materials, the degradation behavior can be tuned by the choice of fabrication strategy, for example, the use of different solvents during processing, or by the incorporation of enzyme-sensitive peptides or degradation-promoting supplements [81]. To mimic the complexity of natural tissue, engineering approaches are destined to use multiple materials, fabricated in various morphology, together with cells and biologicals to carry out specific functions. Genetic engineering is further used to extend the availability and functionality of different silks for tissue engineering applications. Nagano et al. added a poly(glutamic acid) domain to the repetitive amino acid sequence of *B. mori* fibroin to incorporate calcium-binding sites for mineralization [82]. In another study, Saotome et al. improved revascularization by introducing the vascular endothelial growth factor and the RGD-cell adhesion motif into the silk fibroin heavy chain of transgenic *B. mori* silkworms [83].

The majority of recombinant spider silk proteins for biomedical applications is produced in the heterologous expression system *Escherichia coli*. Therefore, the natural silk sequence is determined first and then engineered to be produced in the host organism. After transformation and production, protein purification yields recombinant spider silk proteins [78]. Most recombinant sequences are based on proteins of the major ampullate silks from either *Nephila clavipes* or *Araneus diadematus* spiders [84]. For example, a chimeric protein was genetically engineered containing a consensus sequence from *N. clavipes’* dragline silk fused to the carboxyl-terminal domain of the dentin matrix protein 1 [85]. In another work, Gomes et al. created a fusion protein by combining a consensus sequence from *N. clavipes’* dragline silk with the complete sequence of bone sialoprotein for improved cell attachment and deposition of calcium phosphate [71]. Cellular adhesion and proliferation were enhanced on materials made of an *A. diadematus* derived recombinant spider silk protein fused with the cell adhesion motif RGD [68]. Alternatively, mineral- and collagen-binding motifs were introduced to this protein for materials applications at the tendon-bone interface [72].

### 4.3. Silk-Based Morphologies

Naturally derived or bioengineered silk proteins can be processed into various morphologies. *B. mori* fibroin films are obtained by dissolving the proteins in aqueous lithium bromide solutions and dialysis against water, followed by film casting. To obtain insoluble films, they can be treated with a mixture of water and methanol [86,87]. Similarly, recombinant spider silk proteins are processed into films by dissolving them in the organic solvent hexafluoroisopropanol (HFIP), followed by casting and post-treatment with isopropanol or methanol [88,89].

To imitate the natural fibrous ECM more closely for tissue engineering applications, non-woven mats containing fibroin or spider silk fibers have been produced using electrospinning. The technique constantly evolved to gain more control over the process and outcome. Traditionally, *B. mori* fibroin is dissolved in organic solvents like HFIP, hexafluoroacetone (HFA) or formic acid and spun by applying voltages between 2 kV to 30 kV. Non-woven mats containing fibers with diameters in the low nanometer range up to one micrometer were generated with this set-up [90,91]. In a recent work by Keirouz et al. [92], composite fibers were spun using nozzle-free electrospinning. DeSimone et al. [93] developed an all aqueous electrospinning process for recombinant spider silk proteins. The elimination of harsh processing conditions led to the conformational stability of biological components throughout spinning and posttreatment, promising the inclusion of sensitive biological components for tissue engineering applications [93]. Upon blending poly(caprolactone) (PCL) with poly(glycerol sebacate) and silk fibroin, also the hydrophilicity of the non-woven mats could be increased, which is beneficial for tissue engineering applications [94].

Nano- and microparticles are used in tissue engineering within three-dimensional scaffolds, e.g., to introduce biologically or chemically active factors in constructs or to increase the mechanical stability. Silk particles can be produced by salting-out with potassium phosphate. Tuning protein concentration and mixing intensity, particles in the size range between 150 nm and 10 µm can be fabricated [95,96]. Fibroin particles with a diameter of around 6 µm can also be produced by chopping and wet-milling *B. mori* fibers, while even smaller particles, down to 200 nm, were produced using ethanol precipitation and freezing [97,98]. Through well-suited loading and release properties for various substances, silk particles can be applied as drug carriers. For example, a human recombinant bone morphogenic protein has been successfully encapsulated in silk fibroin particles allowing sustained delivery thereof in bone tissue engineering approaches [69]. Furthermore, the recombinant nature of spider silk proteins allowed genetic modification for covalent, triggerable substance delivery systems [99].

For top-down tissue engineering approaches, porous three-dimensional structures offer adherence points and mechanical stability for cells, and pores facilitate nutrient, oxygen and waste transport. Foaming of silk fibroin solutions with varying nitrous oxide pressure and protein concentration led to scaffolds with pore sizes in the range between 100 and 400 µm [100]. Pore sizes below 100 µm were generated by increasing the protein concentration from 5 wt.% up to 12 wt.%, followed by freeze-drying and immersion in methanol [101]. Recombinant spider silk proteins have been processed into scaffolds with pores sizes of around 100 µm by dissolving the protein in HFIP and using different sized salt crystals as porogens [102].

Hydrogels are hydrophilic polymer networks, physically or chemically cross-linked, that can absorb water up to thousands of times their dry weight [103]. Recombinant spider silk proteins form hydrogels through chain entanglement, which can be printed using dispense plotting at room temperature while supporting encapsulated cells [104]. Due to unfavorable physical properties, like low viscosity, hydrogels made of *B. mori* silk fibroin are less suitable for bioprinting applications without additives. Here, strategies to blend the material with other (bio)polymers to enhance printability have been applied. For example, Chameettachal et al. successfully bioprinted fibroin-gelatin-blends using dispense plotting at room temperature [105].

## 5. Silk-Based Hard Tissue Engineering

The following chapter summarizes recent approaches in silk-based hard tissue engineering, and in Table 1, various examples are listed. Upon providing multiple examples for hard tissue engineering approaches based on silk, the adaptability and compatibility of silk materials are shown.

### 5.1. Bone Tissue Engineering

Among the recent approaches in hard tissue engineering for various tissue types (bone, tendon, cartilage), three types of scaffold materials are used. On the one hand, there are studies based on non-mineralized scaffolds, which were examined concerning their biocompatibility and properties for bone repair without pre-mineralization. Others were mineralized upon incubation with cells or mineralization agents. In the third set-up, inorganic components such as bioceramics or minerals were directly added to the fabrication process to yield composite scaffolds. The following section is describing these three types in more detail, as well as the morphology of the underlying silk scaffolds.

#### 5.1.1. Non-Mineralized Scaffolds

Scaffolds can be fabricated in different dimensions, from 1D fibers, to 2D films to 3D printed scaffolds. Different processing methods have been used to influence the mechanical properties of the silk scaffolds to be more bone-like and to adopt its performance in cell culture. Silk fibroin films as 2D structures were blended with glycerol and poly(ethylene glycol) (PEG) to improve ductility and porosity, beneficial for cell adhesion. Different film properties were obtained upon adjusting the casting temperature. For example, the ultimate tensile strength was increased when films were cast at 60 °C in comparison to 4 °C, indicating higher crystallinity in the films. Moreover, pore sizes decreased in the same manner when film casting was conducted at elevated temperatures. Further, double blends with PEG and glycerol showed the best results, as both additives might interact, yielding stable constructs [106].

Hard tissue repair has high demands regarding the form of the construct, as defect sites are individual and often complex and, therefore, 3D structures such as foams, sponges, injectable or printable hydrogels are of most interest. Silk scaffolds with no chemical crosslinker were fabricated using a solvent exchange method. Silk fibroin as well as spider silk proteins were dissolved in formic acid or HFIP and blended with sodium chloride crystals as porogens to control pore sizes between 200–300 µm. The obtained structures showed high content of β-sheet structures resulting in stable constructs [116]. 3D spongy silk/sericin scaffolds were fabricated using freeze-drying in order to investigate the influence of sericin addition to the material. Structural, biological and immunological properties were investigated with different weight ratios of sericin (0–4.7 wt.%). Further, scaffolds were chemically crosslinked using glutaraldehyde vapor. Structural transition towards β-sheets was induced upon immersion in ethanol. These highly porous structures with more than 90% porosity showed a decrease in pore size in the presence of increasing amounts of sericin. Similar trends were observed for mechanical properties, which were significantly higher upon increasing sericin content. As a result, cell culture studies with human osteoblast MG63 cells revealed no enhanced cytotoxic effect of the sericin present in the scaffolds. Further, macrophage adhesion was not highly pronounced, and inflammatory marker genes were not upregulated with increasing sericin content [117]. Another scaffold was fabricated using lyophilization to investigate the effect of pre-seeding of human adipose-derived mesenchymal stem cells for bone regeneration in vitro and in vivo [107]. Harsh crosslinking agents could be avoided, and constructs were solely post-treated in ethanol. In cell studies, cytocompatibility was confirmed, and a mineralized matrix formation was found after two weeks as a sign of osteogenic differentiation [107]. Rat calvaria models served to evaluate the in vivo performance of cell-seeded scaffolds in comparison to that of non-seeded silk scaffolds. Micro-CT showed no significant impact on the amount of regenerated bone after 12 weeks [107]. However, looking deeper into the composition of the newly formed tissue, new bone with a higher amount of collagen and vasculature was formed in pre-seeded scaffolds [107].

**Figure 4:**
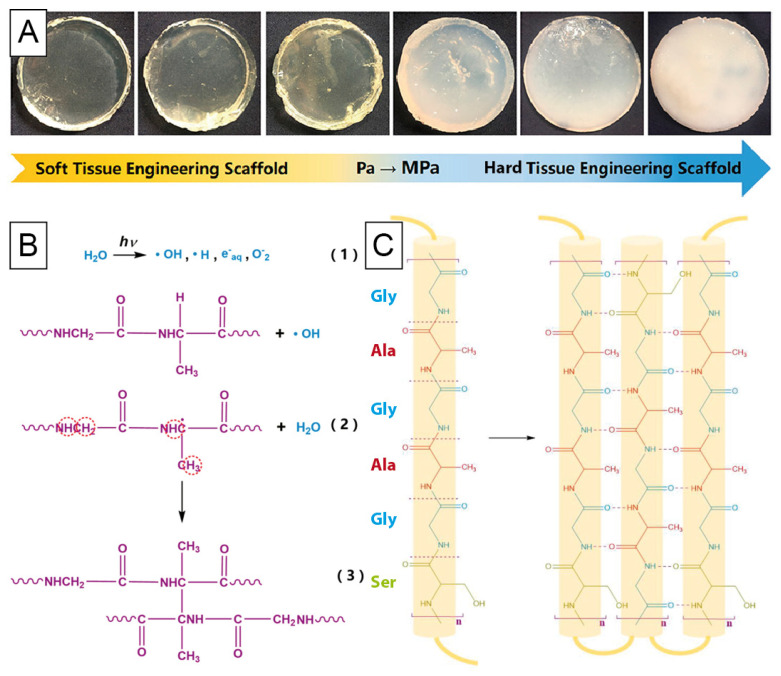
(**A**) Photographs of *Bombyx mori* silk fibroin hydrogels with a controlled degree of crosslinking using γ-radiation (yellow) and ethanol treatment (blue) to cover six orders of magnitude of material stiffness. Crosslinking mechanism upon (**B**) γ-radiation, triggering radical water splitting and the evolution of free radicals, leading to combinational events between polymer chains and (**C**) ethanol treatment, inducing hydrogen bonding and intermolecular interaction. Reprinted and adopted with permission from ref. [119]. Copyright 2020 American Chemical Society.

When using hydrogels for hard tissue engineering, mechanical properties must be investigated and adapted. Different crosslinking methods were used in the following three approaches: Long et al. [118] mixed silk fibroin and elastin to assemble hybrid hydrogels using a physical heat crosslinking method for higher β-sheet content in the silk material and chemical crosslinking with glutaraldehyde between silk and elastin. In this case, silk was used due to its easy processing into different morphologies and elastin to add biochemical cues to the material. Hydrogels exhibited 4–70 kPa in compressive modulus and shear compressive moduli up to 40 kPa. A proliferation assay using L929 lung fibroblasts showed no negative effects of the chemical crosslinking. Wu et al. [119] also combined two crosslinking methods for silk fibroin hydrogels, but in this case, ethanol for physical and γ-ray for chemical crosslinking of the solutions were applied. Crosslinking through irradiation occurred with the assumed mechanism of radical formation as a result of high energy transfer to silk fibroin molecules. Such treated hydrogels could cover several orders of magnitude in elastic moduli from the Pa range up to hard hydrogels in the MPa range upon different irradiation times (Figure 4). Reflecting the possible variation of mechanical properties, irradiation did also alter pore structure, resulting in denser gels with smaller pores upon increasing crosslinking. Interestingly, biodegradation in the presence of protease XIV was not influenced upon irradiation but upon ethanol physical crosslinking, as the latter is related to silk crystallinity. Cell toxicity of rat bone marrow mesenchymal stem cells was studied using supernatants from scaffolds, as irradiation might cause toxic radicals in solution, but no significant changes compared to the control group were observed.

Laomeephol et al. [120] used a phospholipid (1,2-dimyristoyl-sn-glycero-3-phospho-(1′-rac-glycerol) sodium salt) as a gelling additive to accelerate hydrogel formation of silk fibroin solutions. Changing the concentration of the lipid resulted in different gelation rates, and, thereby, gelation could be controlled. The mechanism is based on the amphiphilic nature of the lipid, which forms electrostatic and hydrophobic interactions with silk fibroin. Cytocompatibility was confirmed using ISO 109931:2009 cell tests and cell lines such as L929 lung fibroblast and NIH/3T3 fibroblasts [120]. While proliferation was found in the gels using these cell lines, cancer-derived cells such as SaOS-2 did not proliferate after 21 days and stayed in round shape. In this case, the described constructs seemed to be rather unfavorable substrates while showing inhibition of growth of cancer-derived cell lines [120].

To use silk hydrogels in 3D printing applications, one blend was examined using silk fibroin and alginate [121]. Since bioinks further contain cells for 3D printing applications, they must be cytocompatible and printable to enable cell survival and, in the best case, proliferation. As shear forces are acting on the material during printing, cells have to be protected from damaging shear stress. In this study, 1 wt.% sodium alginate with 2 wt.% silk fibroin underwent rapid gelation upon addition of calcium chloride, which crosslinks alginate [121]. The addition of 1 wt.% rather than 0.5 wt.% alginate resulted in higher strand fidelity during printing. Encapsulated osteosarcoma cells were loaded into the hydrogel blends and manually printed from syringes, which represents a first printability evaluation of a material. Extruded strands were cultured, and cell viability was evaluated in live/dead staining after print-induced shearing. Besides few dead cells in one sample, cells survived the process. Cell viability was confirmed using the metabolic PrestoBlue assay. However, it was found that at higher amounts of silk fibroin, less signal could be detected, although viable cells were found. This led to the assumption that the dye, but also nutrients and waste products can only hardly travel through the construct upon increasing silk fibroin content.

For the adjustment of mechanical properties and printability, filler materials such as nanocellulose can be added to silk fibroin hydrogels [108]. Bacterial nanocellulose was added, yielding composite hydrogels for 3D printing. Photo-crosslinking using tris(bipyridine)ruthenium(II) chloride as crosslinker generated scaffolds, which differed in their characteristics depending on the morphology of the nanocellulose used either as a solution, fibers or whiskers. Structural information was related to β-sheet content and silk fibroin nanocellulose interaction, which is defined by inter-domain distance in the silk fibroin determined using small-angle neutron scattering techniques. Scanning electron microscopy images (SEM) showed that cellulose as an additive influenced pore sizes, yielding especially dense structures in the presence of whiskers. Best rheological, tensile and compression behavior was found in the presence of fiber fillers. Printability evaluated by strand-width after printing was similarly high for fiber and whisker additives. However, as a small drawback, these two morphologies were not reported to be cell friendly whilst causing cytotoxicity upon oxidative stress or inflammatory response. Culturing L929 lung fibroblasts on hydrogels, all blends showed proliferation and high viability tested after 1, 3 and 5 days.

**Figure 5:**
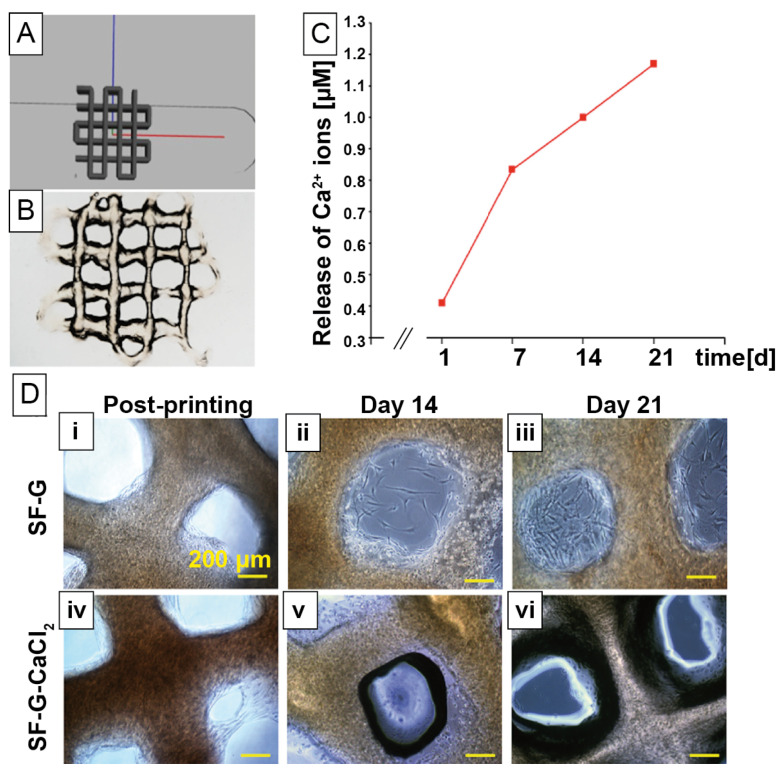
(**A**) CAD sketch of a *Bombyx mori* silk fibroin/gelatine construct and (**B**) its 3D dispense plotted result. (**C**) Release profile of calcium ions from *Bombyx mori* silk fibroin/gelatine hydrogels, loaded with calcium chloride. (**D**) Light microscopy images of unloaded (**i**–**iii**) and loaded (**iv**–**vi**) silk/gelatine hydrogels with human mesenchymal stem cells post-printing (**i**,**iv**), after 14 (**ii**,**v**) and 21 days (**iii**,**vi**). Unloaded scaffolds showed cells present in both the construct and in pores, whereas, in the loaded construct, cells remained in the strands. SF = silk fibroin, G = gelatine. Reprinted and adopted with permission from ref. [122]. Copyright 2019 American Chemical Society.

In another approach, tyrosinase-crosslinked silk fibroin/gelatine hydrogel blends were cell loaded and 3D printed to gain functional constructs [122]. Silk was functionalized with gelatine and calcium chloride for sustained release of calcium ions from the scaffold, similar to the extracellular release of calcium by osteoclasts during bone modeling (Figure 5). Several aims were followed in this study: First, the material was optimized for 3D printing concerning long-term stability for cell culture, and, therefore, rheological properties were adjusted. It was found that at low shear rates, blend hydrogels showed shear thickening behavior, presumably related to crystallization or entanglement between both components. At higher shear rates, a sudden transition to shear thinning behavior was assumed as a result of compound release. Further, the addition of calcium chloride increased the viscosity in such hydrogel blends up to 100-fold due to faster gelation times and ionic interactions with silk fibroin. Second, the osteogenic profile of the constructs was investigated upon release of calcium ions. Ion release was found for 3 weeks, but the release was not complete at that time point.

Third, the signaling pathway, which regulates osteogenic differentiation in human bone marrow derived progenitor cells, was analyzed with regard to the influence of calcium ions. The gene expression profile of parts of the canonical Wnt pathway with specific expression of β-catenin, BMP2 and BMP4 was investigated, and their concentration was highest on day 21. It can be therefore assumed that BMP plays an important role in osteogenic differentiation of human bone marrow derived progenitor cells.

The recombinant spider silk protein eADF4(C16), which is based on the consensus sequence of one component of the *A. diadematus* dragline silk [123], was found to form hydrogels in a controlled manner forming a physical fibrillar network [124,125], enabling its processing via 3D printing for tissue engineering [126]. Further, this recombinant spider silk protein could be modified to comprise the well-known RGD cell-binding motif [126]. Modifications of the processing technique, such as creating blends [127,128], incorporation of silica particles [129] or the release of biologicals from hydrogels [130], were investigated and successfully yielded stable gels.

Silk fibroin hybrid materials with two different silk morphologies were studied, for example, by Ding et al. [131] β-sheet rich silk fibroin nanofibers were encapsulated in an amorphous silk matrix. Upon electric field exposition of 50 V for 30 min, nanofibers traveled through the amorphous matrix and aligned. Scaffolds were crosslinked using horseradish peroxidase and yielded anisotropic scaffolds with up to 120 kPa in stiffness. In in vitro studies, bone marrow mesenchymal stem cells showed osteogenic behavior, and an ectopic in vivo bone model was used to investigate osteogenic properties in rate femurs. Between week 8 to 12, newly formed bone was found in the case of the stiffest hydrogel samples, whereas the aligned fiber structure also led to alignment in the new tissue. The fabrication method with two silk fibroin morphologies and electric field alignment was then used by the same group to fabricate gradient hydrogels [132]. In further detail, horseradish crosslinking times and resulting gradient mechanical properties were investigated. The distribution of β-sheet rich silk fibroin nanofibers in the scaffolds also led not only to changed material stiffness but also to gradient pore structures resembling that of native tissue. In cell culture studies, the construct’s properties translated into tissue-specific differentiation of bone marrow mesenchymal stem cells, whereas the soft part induced chondro-related genes, gradually triggering bone formation towards the stiffer end of the scaffold, which was also confirmed in in vivo studies.

In another double silk approach, Liu et al. [133] used silk fibroin solutions, which were first autoclaved to induce nanoparticle formation (in the range of 50–300 nm), then embedded in silk fibroin solutions, followed by freeze-drying to form 3D sponges. To stabilize structures in an aqueous environment, low-molecular weight PEG solutions were used to induce β-sheet formation. Nanoparticles could be extracted, yielding cavities and pores in the scaffold. This generated pore structure introduced permeability and flexibility compared to silk sponges without particle loading. After methanol annealing, better cell adhesion, distribution and growth on scaffolds were observed. In general, particle-loaded constructs, especially with bioceramics, have the potential to support bone tissue engineering due to their pre-mineralization, which is discussed in detail in the section below.

#### 5.1.2. Microcarriers for Bone Tissue Engineering

**Figure 6:**
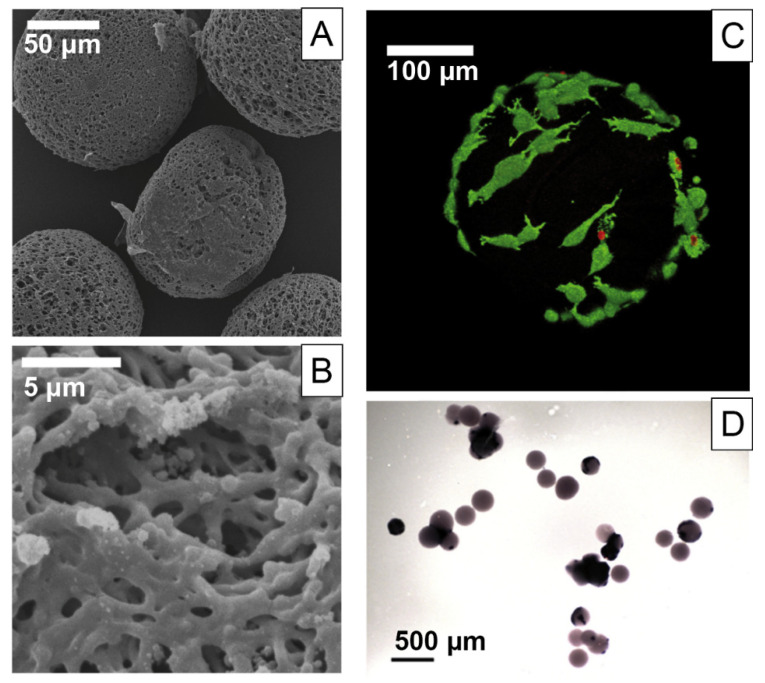
(**A**) Scanning electron microscopy images of blend hydrogel microcarriers using *Bombyx mori* silk fibroin and porcine gelatine at a 3:1 ratio and asymmetric flow focusing in a microfluidic preparation method and (**B**) surface micro-topography at higher magnification. (**C**) Rat mesenchymal stem cells on hydrogel microcarriers visualized using confocal microscopy after 96 h in live/dead staining (dead cells in red, living cells in green) and (**D**) after 28 days. Purple coloring indicates alkaline phosphatase activity of differentiated osteoblasts. Reprinted and adopted with permission from ref. [134]. Copyright 2020 Elsevier.

Microcarriers based on silk fibroin and gelatine were fabricated in a top-down approach for bone tissue engineering as injectable units or building blocks for scaffolds (Figure 6) [134]. With a microfluidic asymmetric flow-focusing device, carriers of about 100–350 µm were produced depending on the flow rate ratio of the aqueous and separation oil phase also containing methanol. The material blend was tested as 2D films and 3D microcarriers in rat mesenchymal stem cell culture. With increasing proportion of gelation from 25 to 50 to 75 wt.%, increasing cell adhesion was found. This trend was similarly confirmed for the carrier’s mechanical properties, as both higher blend situations were in the range of 183 kPa and 139 kPa, respectively, values which are also described for the osteoid region, where pre-osteoblast differentiation takes place.

#### 5.1.3. Biomineralized Scaffolds Using Specific Mineralization Tags

**Figure 7:**
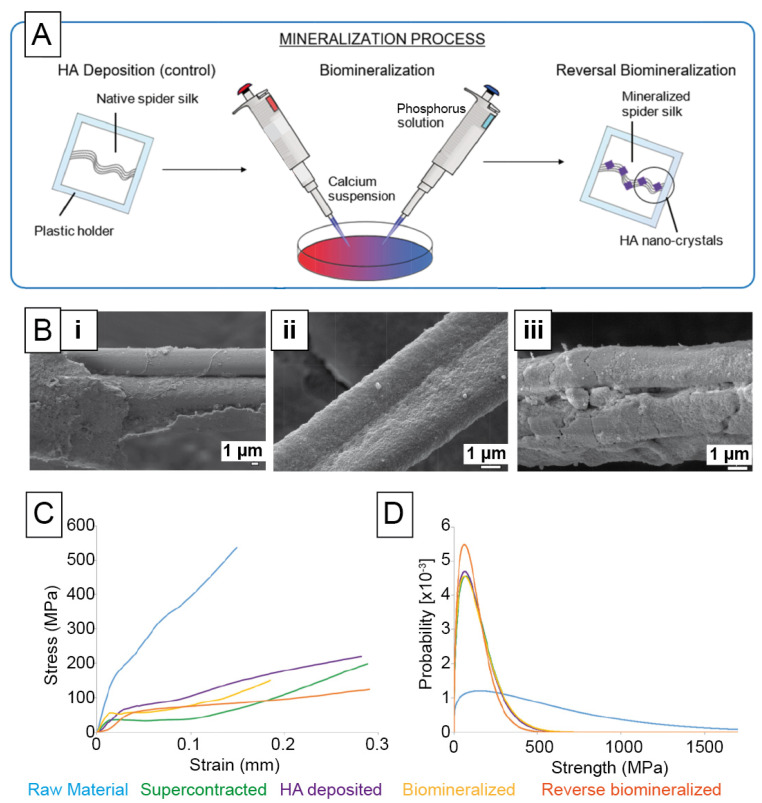
(**A**) Schematic illustration of the mineralization process of natural *Cupiennius salei* spider silk fibers. (**B**) Scanning electron microscopy images of mineralized silk fibers after (**i**) 1 day of biomineralization and day 3 (**ii**) and 7 (**iii**) of reversal biomineralization. Mechanical characterization of fibers in (**C**) stress-strain plots and (**D**) Weibull probability distribution as parameter for homogenous fracture behavior. HA = hydroxyapatite. Reprinted and adopted with permission from ref. [110]. Copyright 2020 John Wiley and Sons.

As a template for biomineralization, natural spider silk fibers were collected from adult females of *Cupiennius salei*. Biomineralization of dragline silk fibers took place upon subsequent incubation in calcium hydroxide containing solution, followed by incubation in diluted phosphoric acid. The procedure was also used the other way round as reversed biomineralization, and further, both solutions were incubated on the fibers simultaneously (Figure 7). Biomimetic hybrid materials were yielded with controlled hydroxyapatite deposition, forming a homogenous coating on the fibers. The best mineralization results were obtained upon initial incubation in calcium-containing solutions, as silk fibers were assumed to interact with the cations and induce higher mineralization with less calcium-deficient hydroxyapatite. Mechanical characterization of the mineralized fibers showed similar strength, toughness and Young’s Modulus in comparison to the natural supercontracted fibers [110].

Smaller fibers in the sub-micron range were produced using electrospinning of *B. mori* silk fibroin out of formic acid and HFIP mixtures into an ethanol bath for instant crosslinking by β-sheet formation. Freeze-drying of the multilayer yielded a 3D fibrous scaffold, and biomineralization was induced upon incubation in two-fold Simulated Body Fluid for up to 28 days. Imaging of the interconnected pores and the increase in fiber diameter allowed an estimation of the ongoing mineral deposition. The effect of the mineralized layers was investigated in vitro using bone marrow mesenchymal stem cells (BMSC) and in vivo in rat cranial defect models. More migrated cells next to the newly formed bone and capillaries confirmed bone regeneration ability of these biomimetic scaffolds [135]. Strong, ductile and lightweight materials were gained upon self-assembly of silk fibroin nanofibers from an aqueous solution. In further processing steps, biomineralization was initiated out of calcium chloride and sodium dihydrogen phosphate solutions before chitin nanofibers were introduced in a hierarchical assembly. Mechanical characterization of the scaffolds revealed a very lightweight material like aerogels but with high compressive strength of up to more than 400 MPa [136].

Further, biomineralization was directed through silk components. Therefore, silk extracted sericin was added to dense collagen hydrogels. Due to the sericin’s negative charge resulting from amino acid residues such as aspartic and glutamic acid, hydroxyapatite formation could be induced. This acellular mineralization process in Simulated Body Fluid yielded minerals after 3 days, with an ongoing process until 14 days resulting in 90 wt.% mineral phase. SEM, energy-dispersive X-ray spectroscopy and X-ray diffraction studies showed distinct spherulite particles. Mesenchymal stem cells were seeded on mineralized sericin-containing collagen hydrogels, and an osteogenic upregulation was observed in metabolic activity [137].

However, biotechnology allows for tailoring recombinant peptides and proteins, and silk proteins were combined with explicit peptide tags on the DNA level to trigger mineralization processes. Engineered sequences from *N. clavipes* dragline silk MaSp1 were C- or/and N-terminally hybridized with the hydroxyapatite binding peptide VTKHLNQISQSY, which was identified via phage display. Films processed out of these proteins were immersed in calcium chloride and sodium phosphate solutions. Mineral formation, as well as human mesenchymal stem cell differentiation, were especially enhanced in the case of double functionalized constructs [60]. The same silk consensus sequence was functionalized with a bone sialoprotein motif to introduce non-collagenous moieties. In this case, silk films were mineralized in female mice in vivo. At first, a mild inflammatory response could be observed using flow cytometry and also histology, but after 6 weeks, inflammation markers decreased. Finally, no capsule formation was observed [71].

In another approach, recombinant spider silk fusion proteins were engineered with different mineralization and collagen-binding motifs from non-collagenous proteins in bone. Proteins with N- and C-terminal peptide tags were compared concerning their mineralization ability in Simulated Body Fluid and their interaction with MC3T3 E1 mouse pre-osteoblasts. The variants showed mineralization tendency to a different extent but confirmed the formation of calcium and phosphate-containing species. Studying cell adhesion on materials of the protein variants separately, no significant favor of one variant over the other could be observed. However, when processing two materials into a gradient, cell adhesion towards the collagen-binding motif was clearly favored over the mineralization variant and could be maintained for 21 days. Therefore, these materials are also suitable candidates for applications at the tendon-bone-interface [72].

#### 5.1.4. Biomineralization of Scaffolds Using Pre-Mineralization

In one example, 1–10 wt.% alumina nanoparticles were added to 4 wt.% silk solutions, and this emulsion was then lyophilized [111]. Additional mineralization was achieved upon incubation of the scaffolds in Simulated Body Fluid [61] for 28 days, forming an apatite layer in all constructs. The cell attachment of rabbit adipose-derived stem cells was not significantly changed with varying alumina content as this material was already reported to be bioinert, leading to the assumption that mechanical and structural cues were affected by the particles. Osteogenic upregulation in an initial stage was found starting at day 7 using alkaline phosphatase activity and Alizarin red staining of the cultures.

Another lyophilized scaffold comprised silk fibroin titanium dioxide and fluoridated titanium dioxide nanoparticles [138]. Particles acted as bioceramic reinforcement for compressive load. As the compressive modulus is often related to particle content, calculations and experimental data were collected. Both approaches were in good agreement with each other and showed open honeycomb structures in the constructs with a compressive modulus of up to 1.297 ± 0.175 MPa in the presence of 20 wt.% TiO_2_.

Magnesium oxide nanoparticle-containing scaffolds at 15/20/25 wt.% were fabricated upon electrospinning of silk fibroin and PCL at a 4:1 *w/w* ratio [139]. Increasing amounts of nanoparticles lead to higher fiber diameters, respectively 651 nm/1055 nm/1251 nm, with visibly entrapped particles. At higher amounts of inorganic fillers, water contact angles decreased (below 30°), turning the materials more hydrophilic and favorable for cells. In comparison, no significant differences between particle-loaded fiber meshes were found concerning cytotoxicity for MC3T3 E1 mouse pre-osteoblast cells. The cumulative release of Mg^2+^ from the meshes reached a plateau after about 15 days. The ion release is often related to osteogenic differentiation and was confirmed with extracts from fiber mats after 21 days using both Alizarin Red and alkaline phosphatase staining. In vivo studies in rat calvarial defects revealed a significant enhancement of bone regeneration using nanofibrous membranes loaded with magnesium oxide particles in the twelfth week post-surgery.

As actuation and dynamic cultures are improving bone regeneration, magnetic particles were incorporated inside silk fibroin scaffolds [140]. Scaffolds were soaked in biomineralization solutions, and Ca/P containing species were obtained. Vibrating Sample Magnetometry was used to characterize magnetic properties, especially for further evaluation of proliferation of MC3T3 E1 cells upon stimulation using a magnetic field. In general, cells grew randomly in the absence and clustered in the presence of a magnetic influence.

**Figure 8:**
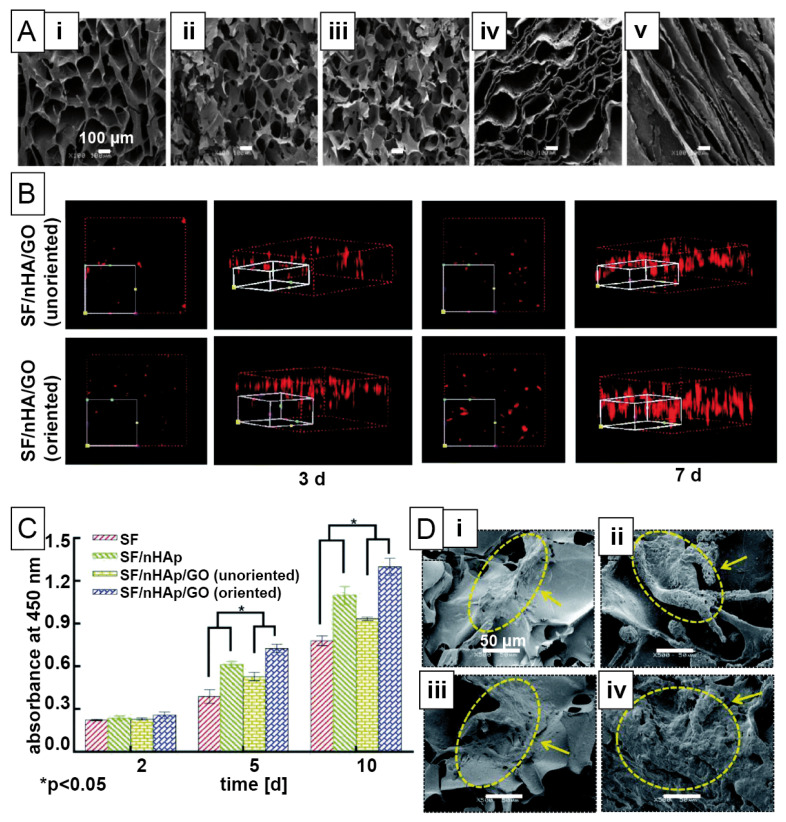
(**A**) Scanning electron microscopy images of lyophilized foam-like scaffolds with different orientation made of plain *Bombyx mori* silk fibroin (**i**), foams loaded with hydroxyapatite nanoparticles (**ii**), foams loaded with hydroxyapatite and graphene oxide nanoparticles in unoriented structures (**iii**), with directional structuring in cross-section (**iv**) and in longitudinal ones (**v**). (**B**) 3D Confocal microscopy images of HUVEC cells in unoriented and oriented foams loaded with hydroxyapatite nanoparticles and graphene oxide nanoparticles after 3 and 7 days. Scale bar not indicated. (**C**) Cell viability assay for bone mesenchymal stem cells after 2, 5 and 10 days. (**D**) Scanning electron microscopy images of bone mesenchymal stem cells in lyophilized foam-like scaffolds after 10 days for (**i**) silk fibroin, (**ii**) foams loaded with hydroxyapatite nanoparticles loaded with hydroxyapatite and graphene oxide nanoparticles without (**iii**) and with (**iv**) directional structuring. SF = silk fibroin, nHA = hydroxyapatite nanoparticles, GO = graphene oxide. Reprinted and adopted with permission from ref. [113]. Copyright 2020 Royal Society of Chemistry.

Graphene oxide has also been added to silk fibroin solutions, followed by lyophilization. In one study, 3D porous silk fibroin/graphene oxide constructs were freeze-dried from solution, and the graphene oxide induced wrinkled surface nanotopographies [141]. Further, its incorporation into scaffolds decreased the diameter of the interconnected pores from 25–60 nm to 10–30 nm. The compressive modulus was independent of the graphene oxide concentration and ranged between 1.5–2 MPa for 3–10 wt.%. Water uptake is a critical factor for cell compatibility and was found to be related to graphene oxide content. Further, water uptake was also crucial for in vitro biomineralization in Simulated Body Fluid. Although mineralization commenced from day 7 to 14, the crystal morphology in the pores changed towards larger and smoother crystals. In a second approach, the synergistic or individual effect of graphene oxide with β-tricalcium phosphate in a lyophilized silk fibroin/soy protein blend scaffold was studied concerning osteoconductivity [112]. Both particle types influenced mechanical properties in the range of 1 MPa. In vitro biomineralization was induced in Simulated Body Fluid for 14 days, and rather small crystals were found covering the whole construct’s surface. Both scaffolds with particles exhibited more minerals than protein blend scaffolds on their own, however, nanocrystal deposition was increased in the presence of graphene oxide only. Alkaline phosphatase activity of rat bone marrow mesenchymal stem cells was highest at day 5, whereas RUNX2 expression as osteogenesis-related gene marker increased until day 14. Osteocalcin expression, as a marker of late osteoblastic differentiation, also increased up to day 14, indicating that the material was suitable for long-term bone regeneration processes. A third filler material was achieved using silk fibroin freeze-dried scaffolds and particles made of nanohydroxyapatite and graphene oxide [113]. Scaffolds with both particle types showed oriented pore structure, similar to lamellae or channels, which was not observed in the other studies. Channel structures were fabricated using a directional temperature field freezing technology, where only one side of the scaffold was exposed to a cold surface and gradually frozen until the fixed structure was freeze-dried. Both oriented and unoriented double-filled materials were fabricated (Figure 8). Mechanical properties were in the kPa range and, therefore, lower than in the two aforementioned studies. Regarding biomineralization, enzyme-directed mineralization was analyzed using alkaline phosphatase activity from bone marrow mesenchymal stem cells. Interestingly, the highest cell viability, and proliferation was found in the case of oriented double-loaded constructs, whereas unoriented constructs showed even lower signals than silk fibroin scaffolds with nanohydroxyapatite only. Besides osteogenic differentiation, the ability was studied to provide structures for vascularization. Human umbilical vein endothelial cells (HUVECs) seeded on the 3D scaffolds migrated preferentially into the aligned channel-like structures as they might sense orientation similar to blood vessels.

For all studies, no phase separation between fillers and silk was observed, indicating good compatibility between the material types. Only at very high filler content, particle aggregates were observed.

### 5.2. Teeth and Mandible Tissue Engineering

State of the art for the regeneration of hard tissue in the mouth is placing implants at the defect location, which need to integrate well at the implantation site. However, it is important to remain the tooth socket for implants, which is rather relating to bone regeneration than teeth themselves. First approaches into this field of application were realized using *B. mori* silk fibroin based scaffolds, prepared via freeze-drying and studied to preserve the jaw ridge [142]: An appropriate rate of material resorption was found for silk fibroin scaffolds with pore sizes around 200 nm and nano-hydroxyapatite reinforcements, which were additionally mineralized in vitro. These scaffolds showed osteogenic differentiation in pre-osteoblast MC3T3 E1 cells after 21 days. Further, the interaction with human bone marrow stromal cells showed good biocompatibility [142].

Another study focused on culturing stem cells from human exfoliated deciduous teeth on silkworm sponges prepared from cocoon cuts. Cell proliferation could be confirmed, but scaffolds for endodontic repair, which can simulate dynamic dental pulp repair, are still at the beginning to commence the field [115].

### 5.3. Tissue Engineering of Bone Neighbouring Hard Tissues

Osteochondral defects can be caused by trauma, tumor resection or osteoporosis and can lead to bone loss, osteoarthritis and even full tissue dysfunction when not treated. Scaffolds used for osteochondral defects often show hierarchical arrangement of chondral and bone bilayers. One example of scaffolds are blends of bacterial cellulose with silk fibroin in an interpenetrating hydrogel to create artificial cartilage [143]. Bacterial cellulose spongy cubes were prepared, squeezed to dry, and silk solutions were soaked in. MC3T3 cell cultures showed the best proliferation on plain silk fibroin, followed by the blended scaffold and finally the cellulose sponge.

Ribeiro et al. [114] crosslinked silk fibroin with horseradish peroxidase as subchrondro-layer and a tricalcium phosphate bone-like layer in a bilayered structure. The scaffolds showed homogenous porosity with macro- and micropore sizes (500 and 10 µm) for the regions with denser structures when mineralized with tricalcium phosphate. Human osteoblasts from femoral bone tissue and human articular chondrocytes were seeded on the scaffolds, and tissue distinct expression patterns were found on the bilayered regions in case of the flexible subchondral and supportive bone region.

**Figure 9:**
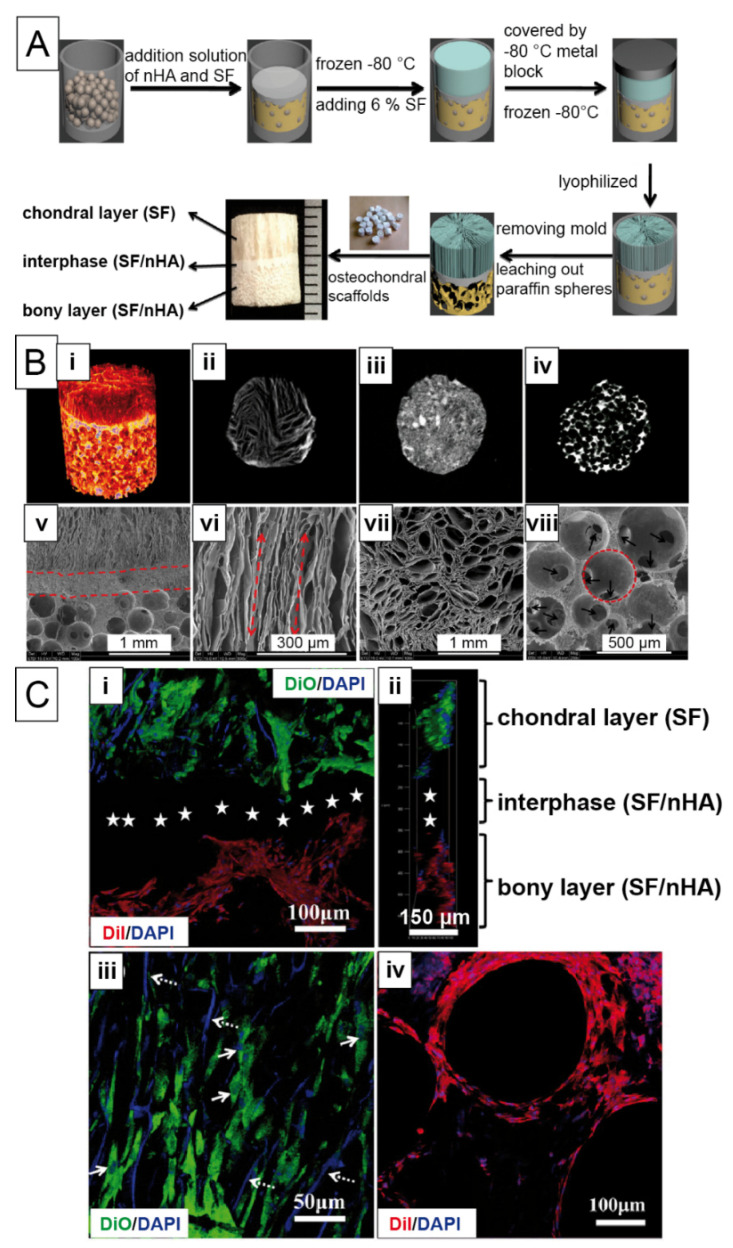
(**A**) Illustration of a trilayer (e.g., chondral, interphase and bony layer) scaffold preparation process using *Bombyx mori* silk. (**B**) Micro-CT (µCT) and scanning electron microscopy (SEM) images of the individual layers in the scaffold: (**i**) µCT of the full construct, (**ii**) µCT of the chondral layer (top), (**iii**) interphase, (**iv**) bony layer (bottom), and (**v**) SEM of a longitudinal section of the full construct (red lines indicate interphases), (**vi**) of a longitudinal section of the chondral layer (red arrows indicate orientation), (**vii**) of cross-sections of the chondral layer, (**viii**) of cross-sections of the bony layer (red circle indicate a macropore and black arrows connections between pores). (**C**) Confocal microscopy images of trilayer scaffolds of rabbit adipose-derived stroma cells after 3 d in (**i**) longitudinal section and (**ii**) profile view with magnifications of (**iii**) the chondral layer and (**iv**) the bony layer. Blue staining indicates cell nuclei (DAPI), green and red staining cell membranes (DiO, DiI) confirming the interphase as an isolation layer. White solid arrows indicate stained cell nuclei, white dashed arrows indicate unspecific staining, white stars indicate the cell-free intermediate layer. SF = silk fibroin, nHA = hydroxyapatite nanoparticles. Reprinted and adopted with permission from ref. [144]. Copyright 2014 American Chemical Society.

In a trilayered scaffold, a cartilage layer, a calcified transition layer and a bony layer were generated upon the addition of nanohydroxyapatite and paraffin-spheres [144]. First, hydroxyapatite in silk solution with paraffin was cast and frozen. Then, on top of the construct, the silk solution was applied and exposed to a cold cylinder using a temperature gradient-guided thermal-induced phase separation technique. After leaching of the paraffin spheres, a lamellar structure in the chondral part, round cavities in the bony part, and an intermediate layer were formed (Figure 9). Adipose-derived stromal cells were seeded on the scaffolds, and both sides had cell favorable structures with lamellae, round pores or cavities. Upon induction of differentiation using respective chondral and bone factors, glycosaminoglycans and collagen type II were found in oriented structures indicating chondrogenic differentiation. In the same manner, bone-related matrix content was found, such as calcium and collagen type I in bone pore structures.

Having confirmed to be able to control differentiation towards the desired tissues based on structural cues, these scaffolds were then applied for rabbit bone repair in the knee, with and without pre-cell seeding [145]. Defect evaluation took place 4, 8 and 12 weeks after implantation. At all points of evaluation, surface roughness and integrity, bone smoothness and genetic upregulation were higher when cells were already present in the scaffold. However, neither bone strength nor quality was affected thereby.

Neighboring tissues towards bone such as tendon or cartilage can also exhibit mineralized regions in a gradual manner. This gradual change in composition and mechanical properties hinders crack propagation and allows a uniform transmission of loads. Qian et al. [109] fabricated structures from collagen type I and silk fibroin with increasingly aligned structures, generated using unidirectional freezing for application at the tendon-to-bone interface. Aligned collagen structures with knitted silk fibers exhibited the highest order. Implantation into rats showed that rather unoriented structures were favored for bone repair, whereas aligned structures triggered tendon regeneration. Therefore, the optimum structural combination still has to be found, as complex processes are interacting at the joint between two tissues.

Bradner et al. [146] fabricated microfibers out of silk fibroin hydrogels with additional functionalization using bovine serumalbumin and a bio-silica precursor peptide. Biomineralization is required at the tendon-to-bone insertion to transmit loads on mineralized fibrils. Silk at 5 wt.% and BSA at 0.2 wt.% resembled the natural ratio of collagen-to-elastin in the tendon. Fibers were extruded and enzymatically crosslinked using horseradish peroxidase, followed by thermal post-treatment. Fibers were braided or twisted by hand. Fiber toughness was increased up to 125.4 ± 3.50 Jm^−2^ upon the addition of BSA. An explanation for this behavior could be the presence of additional sacrificial bonds, which break before the structure collapses. Pre-and post-strained silk-BSA samples showed a hierarchy-enabling microstructural rearrangement.

## 6. Outlook

Major biomaterials in the current global orthopedic market are collagen, hydroxyapatite, calcium phosphates, calcium sulfate and hyaluronic acids [147]. While there are no silk-based products in the field of hard tissue engineering available up until now, recent strategies have shown the eligibility of such materials for hard tissue engineering. Tailor-made solutions for patients will enable personalized medicine, and therein individual requirements for defect solutions can be met. However, top-down strategies rely on construct fabrication, and various complex prerequisites need to be approached concerning the choice of material. Biodegradability is thereby of high importance with respect to non-toxic metabolites and material break down along with tissue regeneration. Hence, biomaterials such as silk-based ones are increasingly in the focus of interest for tissue engineering approaches as they are biocompatible, non-toxic and do not evoke a strong immune reaction by the recipient. Additionally, recombinant silks can be genetically fine-tuned and produced biotechnologically in a large scale. The processing of raw silk proteins into a wide range of morphologies such as particles, fibers, foams and hydrogels allows the coverage of scaffold complexity on various hierarchical levels. In hard tissue engineering, construct design relies to a high degree on the fulfillment of biological and mechanical prerequisites, some of which are based on proper mineralization. Biomimetic mineralization of silk scaffolds can be conveyed upon the introduction of binding sites to accumulate ions from the surrounding media. To our knowledge, currently no clinical studies containing silk-based materials are under way for hard tissue engineering. The available tools to modify and process these materials, as well as the presented promising research results, however, are key further developments. For example, silk screws (*B. mori*) applied as orthopedic fixtures, already successful in animal testing, show high potential for clinical trials [148,149]. Especially with the rise of additive manufacturing techniques and the need, as well as the possibility, for personalized scaffolds within hard tissue engineering, silk-based materials might soon take the next step towards an application.

## Figures and Tables

**Table 1:** Overview of silk-based hard tissue engineering approaches.

Mineralization	Silk Source	Filler Materials/Additives	Morphology/Fabrication Technique	Cell Types	Biocompatibility Study	Target Tissue
non-mineralized	*Bombyx mori* silk fibroin [106]	glycerol, PEG	2D film casting	human dermal fibroblasts	in vitro	bone
–	*Bombyx mori* silk fibroin [107]	–	3D porous scaffold/lyophilization	human adipose mesenchymal stem cells	in vitro and in vivo in rat calvarial bone model	bone
–	*Bombyx mori* silk fibroin [108]	bacterial nanocellulose; photo-crosslinker	3D hydrogels/3D printing	mouse lung fibroblasts	in vitro	bone
–	*Bombyx mori* silk fibroin [109]	collagen I	3D scaffold with aligned or knitted fibers/lyophilization	rabbit bone marrow stem cells	in vitro and in vivo in rotator cuff rabbit model	tendon-to-bone transition
biomineralized	recombinant spider silk [72]	–	2D film casting	mouse pre-osteoblasts	in vitro	tendon-to-bone transition
–	*Cupiennius salei* spider silk fibers [110]	–	2.5D fibers/naturally harvested	–	–	bone
pre-mineralized materials	*Bombyx mori* silk fibroin [111]	alumina nanoparticles	3D porous scaffold/lyophilization	rabbit adipose-derived stem cells	in vitro	bone
–	*Bombyx mori* silk fibroin, soy protein [112]	graphene oxide, β-tricalcium phosphate	3D porous scaffold/lyophilization	rat bone marrow stem cells	in vitro	bone
–	*Bombyx mori* silk fibroin [113]	graphene oxide, nano-hydroxyapatite	3D porous scaffold/lyophilization	bone marrow stem cells, human umbilical vein endothelial cells (HUVECs)	in vitro	bone, vasculature
–	*Bombyx mori* silk fibroin [114]	doped β-tricalcium phosphate, crosslinker	3D porous scaffold/lyophilization	human osteoblasts, human articular chondrocytes	in vitro	bone, cartilage
–	*Bombyx mori* silk fibroin [115]	–	3D porous sponges/salt leaching	stem cells from human exfoliated deciduous teeth	in vitro	teeth
